# Necklace of Teeth

**Published:** 1892-11

**Authors:** 


### Editorial, Etc.

NECKLACE OF TEETH.—"The San Francisco Examiner" says: What the queue is to the Chinaman, what the tuft of hair in the middle of his head is to the American Indian, such to the Fiji Islander as giving him the pass to social distinction is the necklace he wears of teeth taken from his victims.

So says Dr. J. M. Whitney, of Honolulu, and he ought to know. For a few dollars he obtained such a necklace from a sailor who had been on a British man-of-war cruising among the Fiji Islands. Many traits of character and disposition are revealed on examining this necklace. In the first place, there are 288 teeth. As each tooth represents a victim the assumption is that this particular chief lived well and dined sumptuously.

Many of the teeth have not attained their full size. This indicates that the owners of the teeth were young and, of course, tender and juicy. All of which goes to show that the cannibal king was of a discriminating mind and knew good food when he saw it.

On the other hand, the presence of some very old-looking teeth augurs that sometimes times were hard and the poor cannibal had to put up with what he could get. This would encourage the virtues of fortitude and self denial.

---

EIGHTEEN hundred pounds of gold goes into people's teeth every year, put there by dentists.

